# *Sarcocystis* spp. in domestic sheep in Kunming City, China: prevalence, morphology, and molecular characteristics

**DOI:** 10.1051/parasite/2017025

**Published:** 2017-08-02

**Authors:** Jun-Jie Hu, Si Huang, Tao Wen, Gerald W. Esch, Yu Liang, Hong-Liang Li

**Affiliations:** 1 School of Biological Sciences, Yunnan University Kunming 650091 PR China; 2 Southeast Asia Biodiversity Research Institute, Chinese Academy of Sciences, Yezin Nay Pyi Taw 05282 Myanmar; 3 Department of Biology, Wake Forest University Winston-Salem NC 27106 USA

**Keywords:** Sheep, *Sarcocystis*, Prevalence, Morphology, Molecular characteristics, China

## Abstract

Sheep (*Ovis aries*) are intermediate hosts for at least six named species of *Sarcocystis*: *S. tenella*, *S. arieticanis*, *S*. *gigantea*, *S. medusiformis*, *S*. *mihoensis*, and *S*. *microps*. Here, only two species, *S*. *tenella* and *S. arieticanis*, were found in 79 of 86 sheep (91.9%) in Kunming, China, based on their morphological characteristics. Four genetic markers, i.e., 18S rRNA gene, 28S rRNA gene, mitochondrial cox1 gene, and ITS-1 region, were sequenced and characterized for the two species of *Sarcocystis*. Sequences of the three former markers for *S. tenella* shared high identities with those of *S*. *capracanis* in goats, i.e., 99.0%, 98.3%, and 93.6%, respectively; the same three marker sequences of *S. arieticanis* shared high identities with those of *S*. *hircicanis* in goats, i.e., 98.5%, 96.5%, and 92.5%, respectively. No sequences in GenBank were found to significantly resemble the ITS-1 regions of *S*. *tenella* and *S. arieticanis*. Identities of the four genetic markers for *S*. *tenella* and *S. arieticanis* were 96.3%, 95.4%, 82.5%, and 66.2%, respectively.

## Abstract (fr)

Les moutons (*Ovis aries*) sont des hôtes intermédiaires pour au moins 6 espèces nommées de *Sarcocystis* : *S. tenella*, *S. arieticanis*, *S. gigantea*, *S. medusiformis*, *S. mihoensis* et *S. microps*. Ici, seules deux espèces, *S. tenella* et *S. arieticanis* ont été trouvées dans 79 (91.9 %) de 86 moutons à Kunming, en Chine, en fonction de leurs caractéristiques morphologiques. Quatre marqueurs génétiques, le gène de l’ARNr 18S, le gène de l’ARNr 28S, le gène mitochondrial cox1 et la région de l’ITS-1 ont été séquencés et caractérisés pour les deux espèces de *Sarcocystis*. Les séquences des trois premiers marqueurs de *S. tenella* partageaient des similarités élevées avec celles de *S. capracanis* chez les chèvres, soit 99.0 %, 98.3 % et 93.6 %, respectivement; les trois mêmes marqueurs de *S. arieticanis* partageaient des identités élevées avec ceux de *S. hircicanis* chez les chèvres, à savoir 98.5 %, 96.5 % et 92.5 %, respectivement. Aucune séquence de GenBank ne ressemble significativement aux régions ITS-1 de *S. tenella* et *S. arieticanis*. Les identités des quatre marqueurs génétiques de *S. tenella* et *S. arieticanis* étaient respectivement de 96.3 %, 95.4 %, 82.5 % et 66.2 %.

## Introduction

*Sarcocystis* spp. are cyst-forming intracellular protozoan parasites with an obligate two-host life cycle between predators as definitive hosts and prey animals as intermediate hosts. Sheep (*Ovis aries*) are intermediate hosts for at least six species, i.e., *S. tenella*, *S. arieticanis*, *S*. *gigantea*, *S. medusiformis*, *S*. *mihoensis*, and *S*. *microps*, which are morphologically differentiated based on their sarcocyst wall ultrastructure. *Sarcocystis tenella* and *S. arieticanis* produce microscopic sarcocysts transmitted by canids, while *S. gigantea* and *S*. *medusiformis* produce macroscopic cysts transmitted by felids [2]. The remaining two species, *S*. *mihoensis* and *S*. *microps* transmitted by canids, are unusual or rare species of *Sarcocystis*; *S. mihoensis*, reported only from Japan, produces macroscopic sarcocysts [17]; however, *S. microps*, reported only once from China, produces microscopic sarcocysts [18]. Natural infections by *Sarcocystis* spp. in domestic sheep have been investigated in various countries throughout the world, with prevalence ranging from 9.0 to 100% depending on the detection methodology [2]. However, the prevalence of *Sarcocystis* spp. in domestic sheep in China is largely unknown.

The ultrastructure of sarcocysts is traditionally a reliable character for clarifying different *Sarcocystis* species in a given intermediate host. However, with more morphologically similar sarcocysts described from different, but closely related intermediate hosts, confusions or disputes have emerged concerning the relationships of these *Sarcocystis* species. For example, *Sarcocystis* spp. in cattle and water buffalo have been regarded as separate species based on host specificity; however, sarcocysts in bobcats (*Felis rufus*), domestic cats (*F. catus*), Florida panthers and cougars (*F*. *concolor*), and cheetahs (*Acinonyx jubatus*) were all identified as *S*. *felis*, on the basis of the morphological similarities [2]. It is therefore an urgent need, even a must, to delineate or reassess descriptions of extant or new species of *Sarcocystis* in different hosts, using different markers for clarifying their relationships. However, only limited molecular sequences for *Sarcocystis* spp. in sheep are presently provided in GenBank.

Therefore, the aims of the present study were (i) to investigate the prevalence of *Sarcocystis* spp. in domestic sheep in China based on the morphological characteristics of the sarcocysts, and (ii) to characterize these species using the 18S rRNA gene (*18S rRNA*), 28S rRNA gene (*28S rRNA*), mitochondrial cox1 gene (*cox1*), and ITS-1 (*ITS-1*) region for clarifying their descriptions.

## Materials and methods

### Morphological observation of sarcocysts

In total, tissues from 86 sheep were examined from an abattoir in Kunming City in China from March to November 2015. From each animal, fresh tissue samples from the esophagus, diaphragm, skeletal muscles, tongue, and heart were examined for sarcocysts. In the laboratory, 0.5 mm pieces of muscle from each collected sample were pressed and squeezed between two glass slides to inspect sarcocysts using stereomicroscopy. Sarcocysts were isolated from muscular fibers using dissecting needles and processed for light microscopy (LM), transmission electron microscopy (TEM), and DNA analysis.

For TEM, sarcocysts were fixed in 2.5% glutaraldehyde in cacodylate buffer (0.1 M, pH 7.4) at 4 °C and post-fixed in 1% osmium tetroxide in the same buffer, then dehydrated in graded alcohols and embedded in epon-araldite mixture. Ultrathin sections were stained with uranyl acetate and lead citrate and then examined using a JEM100-CX transmission electron microscope at 80 kV. For DNA isolation, individual cysts were stored in sterile water at −20 °C prior to processing.

### Molecular characterization

Two individual sarcocysts of each of the *Sarcocystis* species from sheep were subjected to genomic DNA extraction using the phenol/chloroform method after 0.01% proteinase K and 0.25% trypsin digestion. *18S rRNA* was amplified with primer pairs, S1/B [4, 11]; *28S rRNA* was amplified with primer sets KL1/KL3, KL4/KL5b, and KL6/KL2 [12]; and mitochondrial *cox1* was amplified with primer pairs SF1/SR9 [6, 7]; *ITS-1* was amplified with primer pairs SU1F/5.8SR2 [8]. The polymerase chain reaction (PCR) products were purified, cloned, sequenced, and analyzed using the method detailed in a previous paper [9].

## Results

### Prevalence of natural infections

**Figure 1.**
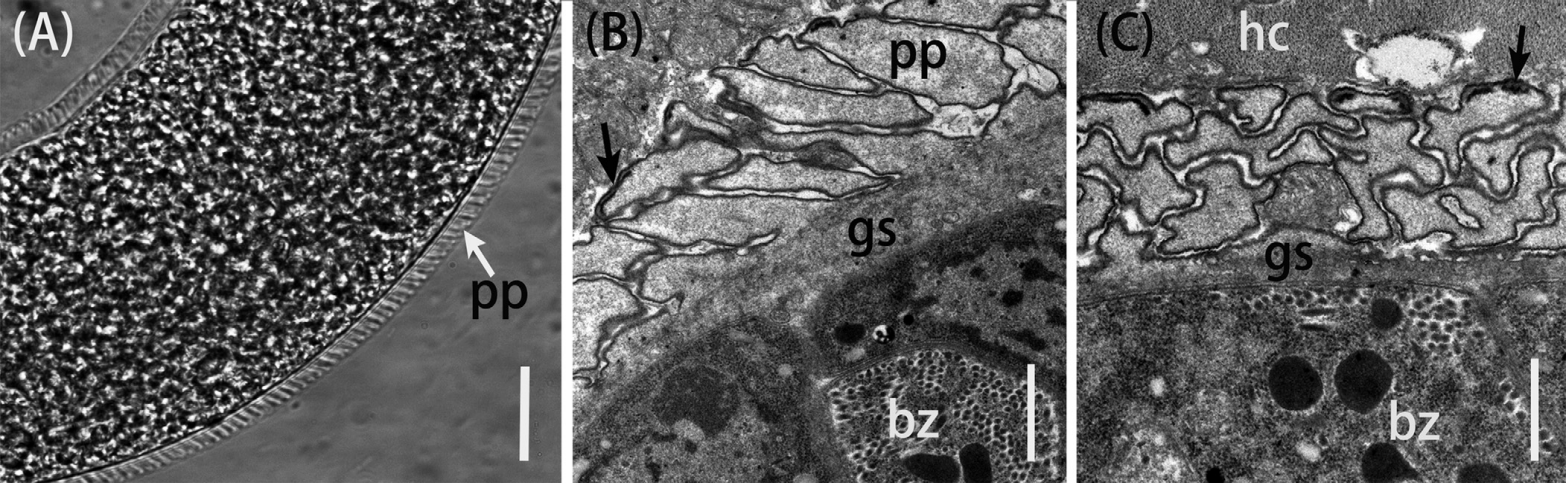
Morphological characteristics of *Sarcocystis tenella* sarcocysts from sheep muscle. (A) Light micrograph of a sarcocyst (unstained); note the sarcocyst wall with numerous palisade-like protrusions (pp). Scale bar = 20 μm. (B) Longitudinal section of sarcocyst wall by transmission electron microscopy (TEM). The sarcocyst wall had palisade-like protrusions (pp), characterized by the apex which contained dense plaques (arrow); a layer of ground substances (gs) located beneath the primary sarcocyst wall surrounded bradyzoites (bz). Scale bar = 1 μm. (C) Cross-section of a sarcocyst by TEM; note the dense plaques (arrow), ground substance (gs), bradyzoites (bz), and host cell (hc). Scale bar = 1 μm.

**Figure 2.**
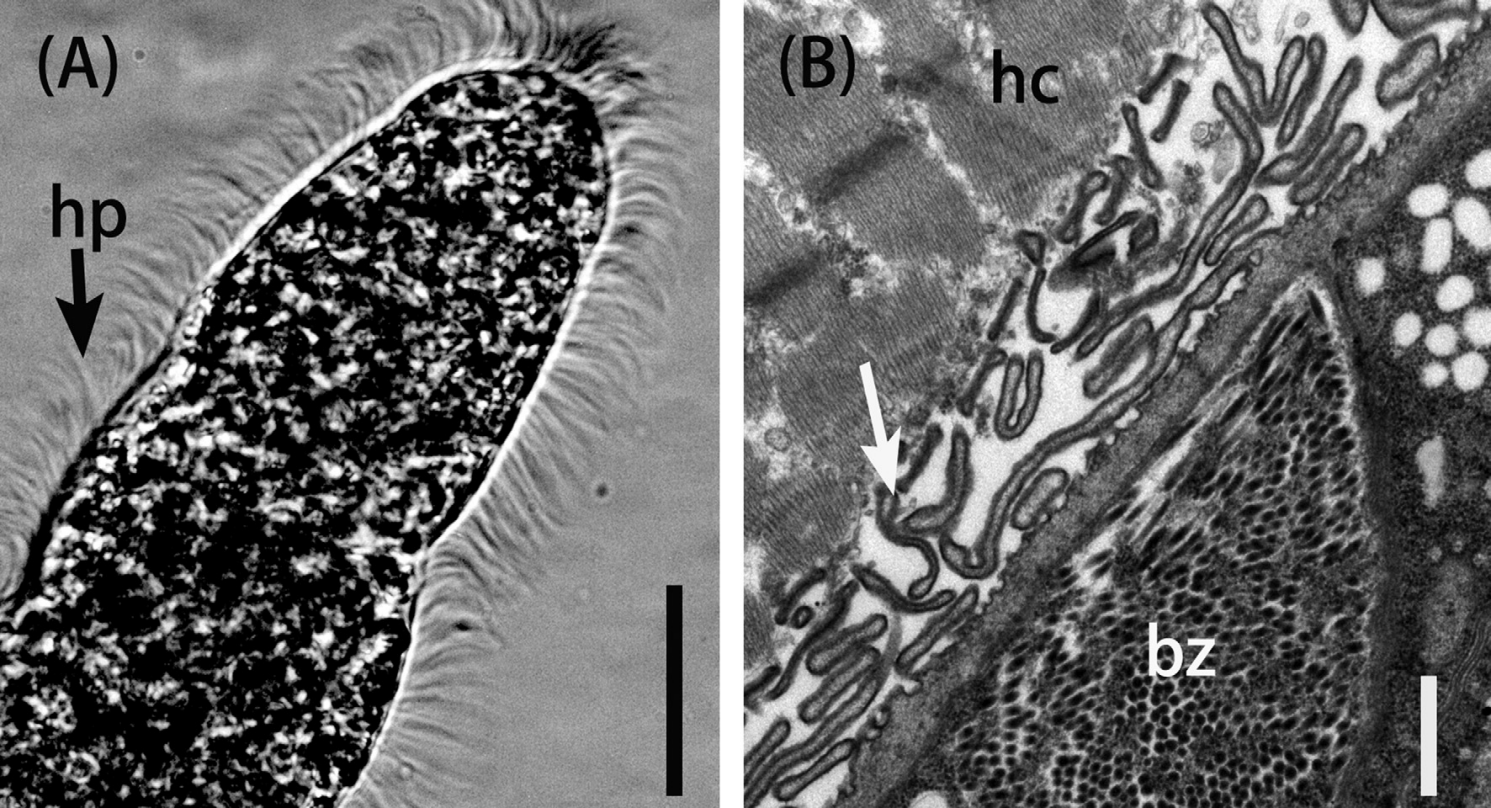
Morphological characteristics of *Sarcocystis arieticanis* sarcocysts from sheep muscle. (A) Light micrograph of a sarcocyst (unstained); note the sarcocyst wall with numerous hair-like protrusions (hp). Scale bar = 20 μm. (B) Transmission electron micrograph of the cyst wall of a sarcocyst; note host cell (hc), bradyzoites (bz), and hair-like protrusions (arrow). Scale bar = 1 μm.

Sarcocysts were found in 79 of 86 sheep (91.9%). Two morphologically distinct sarcocysts (*S. tenella* with a thick cyst wall and *S. arieticanis* with a thin cyst wall) were observed by LM observation (Figs. 1A, 2A). Sarcocysts of *S. tenella* were found in 73 sheep (84.9%), and were more common than those of *S*. *arieticanis*, found in 46 sheep (53.5%). The distribution of the two parasites in different organs is shown in Table 1.

**Table 1.** Prevalence of *Sarcocystis* spp. in different muscular tissues of sheep (*n* = 86) in Kunming, China.

Muscle infected	*Sarcocystis* species					
	*S*. *tenella* and/or *S*. *arieticanis*		*S*. *tenella*		*S*. *arieticanis*	
	No. infected	% infected	No. infected	% infected	No. infected	% infected
Esophagus	73	84.9	73	84.9	44	51.2
Tongue	28	32.6	28	32.6	17	20.0
Diaphragm	49	57.0	48	55.8	21	24.4
Heart	18	20.9	18	20.9	0	0
Skeletal muscles	68	79.1	68	79.1	36	41.9
Total infected animals	79	91.9	73	84.9	46	53.5

### Light and electron microscopy observations of sarcocysts

Using LM, sarcocysts of *S*. *tenella* were observed to be microscopic, measuring 650–1,350 × 65–130 μm (*n* = 30) in size. The sarcocyst wall had numerous, 3.0–5.8 μm (*n* = 30) long, palisade-like protrusions (Fig. 1A). The sarcocysts were septate and their interior compartments were filled with bradyzoites measuring 11.0–14.0 × 3.5–5.0 μm (*n* = 30) in size. By TEM, the sarcocysts had numerous villous or palisade-like protrusions, were 3.1–4.6 × 1.2–1.7 μm (*n* = 10) in size, and were characterized by the apex that contained dense plaques; microtubules were absent (Figs. 1B, 1C). A layer of ground substances measuring 0.4–0.6 μm in thickness was located immediately beneath the primary sarcocyst wall.

Sarcocysts of *S*. *arieticanis* were microscopic, measuring 137–1,272 × 75–103 μm (*n* = 30) in size (Fig. 2A) by LM. The sarcocyst wall had numerous, 4.7–6.0 μm (*n* = 30) in length, hair-like protrusions. The cysts were septate and their interior compartments were filled with bradyzoites measuring 9.5–13.6 × 4.0–5.0 μm (*n* = 30) in size. The ultrastructure of the sarcocysts showed the presence of irregularly folded, but non-branched, hirsute or bone-like protrusions (Fig. 2B). A layer of ground substances measuring 0.3–0.5 μm in thickness was located immediately beneath the primary sarcocyst wall.

### Molecular characterization of the *18S rRNA*

The two *18S rRNA* nucleotide sequences, each from an individual sarcocyst of *S*. *tenella*, were 1,832 bp in length, and completely identical; as a result, only one sequence (MF039329) was submitted to GenBank. The most similar sequences in GenBank were those of *S*. *tenella* (KC209734 and KC209737) from sheep (99.8–99.9% identity, on average 99.9% identity), followed by *S. tenella* (KP263752–KP263759) from chamois (*Rupicapra rupicapra*) (99.7–99.9% identity, on average 99.8% identity), *S*. *capracanis* (L76472, KU820982, and KU820983) from goats (*Capra hircus*) (99.0–99.1% identity, on average 99.0% identity), *S*. *alces* (KF831273 and KF831274) from moose (*Alces alces*) (97.6–97.8% identity, on average 97.6% identity), *S*. *heydorni* (KX057996 and KX057997) from cattle (*Bos taurus*) (97.5–97.6% identity, on average 97.5% identity), *S*. *tarandivulpes* (EF056012) from reindeer (*Rangifer tarandus*) (97.3% identity), and *S*. *cruzi* (JX679468) from cattle (97.0% identity).

The two *18S rRNA* sequences (MF039330 and MF039331), each obtained from an individual sarcocyst of *S*. *arieticanis*, were 1,836 bp in length, and shared 99.9% identity. The differences included three nucleotide substitutions. Identity of the new *18S RNA* sequences between *S*. *arieticanis* and *S*. *tenella* was 96.1–96.2% and 96.2% on average. The most similar sequences in GenBank were those of *S*. *hircicanis* (KU820984 and KU820985) from goats (98.5–98.6% identity, on average 98.5% identity), followed by *S*. *arieticanis* (L24382) from sheep (98.3–98.5% identity, on average 98.4% identity), *S*. *cruzi* (KT901167, JX679467, and JX679468) from cattle (96.5–96.8% identity, on average 96.6% identity), *S*. *hjorti* (JX679468) in moose (96.3–96.4% identity, on average 96.3% identity), *S*. *levinei* (KU247914–KU247922) from water buffalos (*Bubalus bubalis*) (96.4–96.5% identity, on average 96.5% identity), *S*. *pilosa* (KU753891–KU753893) from sika deer (*Cervus nippon*) (96.3–96.4% identity, on average 96.3% identity), and *S*. *tenella* (KC209737) from sheep (96.2–96.3% identity, on average 96.3% identity).

### Molecular characterization of the *28S rRNA*

The two *28S rRNA* nucleotide sequences (MF039325 and MF039326), each obtained from an individual sarcocyst of *S. tenella*, were 3,459 bp in length, and shared 99.8% identity. The differences included six nucleotide substitutions. The most similar sequence in GenBank was that of *S*. *tenella* (AF076899) (98.9–99.1% identity, on average 99.0% identity), followed by *S*. *capracanis* (AF012885, KU820978, and KU820979) (98.2–98.4% identity, on average 98.3% identity), *S*. *cruzi* (AF076903) (95.6–95.8% identity, on average 95.7% identity), *S*. *arieticanis* (AF076904) (95.4–95.5% identity, on average 95.4% identity), and *S. hircicanis* (KU820980 and KU820981) (94.3–95.0% identity, on average 94.6% identity).

The two *28S rRNA* sequences (MF039327 and MF039328), each obtained from an individual sarcocyst of *S. arieticanis*, were 3,482 bp and 3,506 bp in length, respectively, and shared 97.8% identity. The differences included 42 nucleotide substitutions and 23 nucleotide deletions. Identity of the new *28S rRNA* sequences between *S*. *arieticanis* and *S. tenella* was 95.3–96.4%, and 95.9% on average. The most similar *28S rRNA* sequences in GenBank were those of *S*. *arieticanis* (AF076904) (97.6–99.1% identity, on average 98.3% identity), followed by *S. hircicanis* (KU820980 and KU820981) (96.4–96.7% identity, on average 96.5% identity), *S*. *tenella* (AF076899) (96.0–96.8% identity, on average 96.4% identity), *S*. *capracanis* (AF012885, KU820978, and KU820979) (95.7–96.5% identity, on average 96.1% identity), and *S*. *cruzi* (AF076903) (95.0–95.1% identity, on average 95.0% identity).

### Molecular characterization of the mitochondrial *cox1*

The two mitochondrial *cox1* nucleotide sequences (MF039322 and MF039323), each from an individual sarcocyst of *S. tenella*, were 1,038 bp in length, and shared 98.3% identity. The differences included 15 nucleotide substitutions. They shared the highest identities with those of *S*. *tenella* (KC209723–KC209732) from sheep (96.9–97.5%, on average 97.2%) and *S*. *tenella* (KP263744–KP2637451) from chamois (96.9–97.5% identity, on average 97.2% identity), followed by *S*. *capracanis* (KU920974, KU820977) (93.6% identity), and *S*. *heydorni* (KX057994 and KX057995) (90.1–90.3% identity, on average 90.2% identity).

The two mitochondrial *cox1* nucleotide sequences, each obtained from an individual sarcocyst of *S. arieticanis*, were 1,038 bp in length, and shared 100% identity; accordingly, only one sequence (MF039324) was submitted to GenBank. The *cox1* nucleotide sequence identity between those for *S*. *arieticanis* and *S. tenella* was 82.2–82.6%, and 82.4% on average. The most similar *cox1* sequences in GenBank were those of *S*. *hircicanis* (KU820975 and KU8209756) (92.4–92.7% identity, on average 92.5% identity), followed by *S*. *grueneri* (KC209615–KC209624) from reindeer (83.7–84.5% identity, on average 84.2% identity), *S*. *capreolicanis* (KY018939–KY018944) from roe deer (*Capreolus capreolus*) (82.6–82.9% identity, on average 82.8% identity), *S*. *capracanis* (KU820974 and KU820977) (82.5–82.8%, on average 82.6%), and *S*. *tenella* (KC209725 and KC209731) (82.4–82.6% identity, on average 82.5% identity).

### Molecular characterization of the *ITS-1*

The two *ITS-1* nucleotide sequences (MF039318 and MF039319), each from a sarcocyst of *S. tenella*, were 784 bp and 787 bp in length, respectively; the identity between them was 96.7%, and the differences included 23 nucleotide substitutions and three nucleotide deletions. The two *ITS-1* nucleotide sequences (MF039320 and MF039321), each from a sarcocyst of *S. arieticanis*, were 784 bp and 786 bp in length, respectively; the identity between them was 97.0%, and the differences included 20 nucleotide substitutions and four nucleotide deletions. Blast search only using the *ITS-1* region, of approximately 525 bp for *S. tenella* and 520 bp for *S. arieticanis*, revealed that no sequences shared significant similarities with them. The identity of the new *ITS-1* nucleotide sequences for the two species of *Sarcocystis* in sheep was 65.7–69.7%, and a mean of 66.2%.

**Figure 3.**
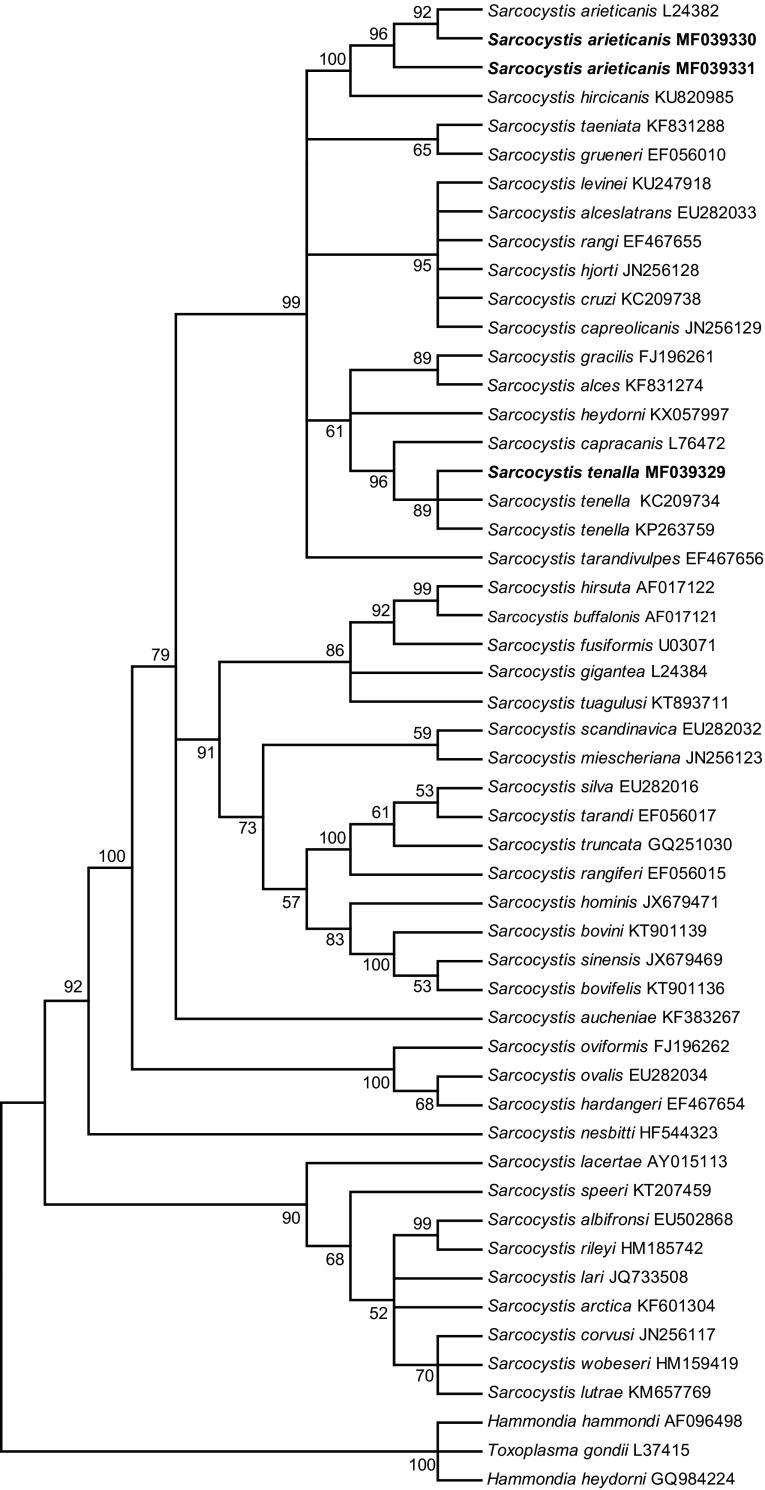
Phylogenetic tree for selected members of the Sarcocystidae based on *18S rRNA* sequences and inferred by the maximum parsimony (MP) method using the Tree-Bisection-Regrafting (TBR) algorithm. The analysis involved 52 nucleotide sequences (GenBank accession numbers behind the taxon names), and a total of 2,014 positions in the final dataset. The values between the branches represent percent bootstrap value per 1,000 replicates, and the values below 50% are not shown. The three new sequences of *Sarcocystis tenella* (MF039329) and *S*. *arieticanis* (MF039330 and MF039331) have the taxon name in boldface.

**Figure 4.**
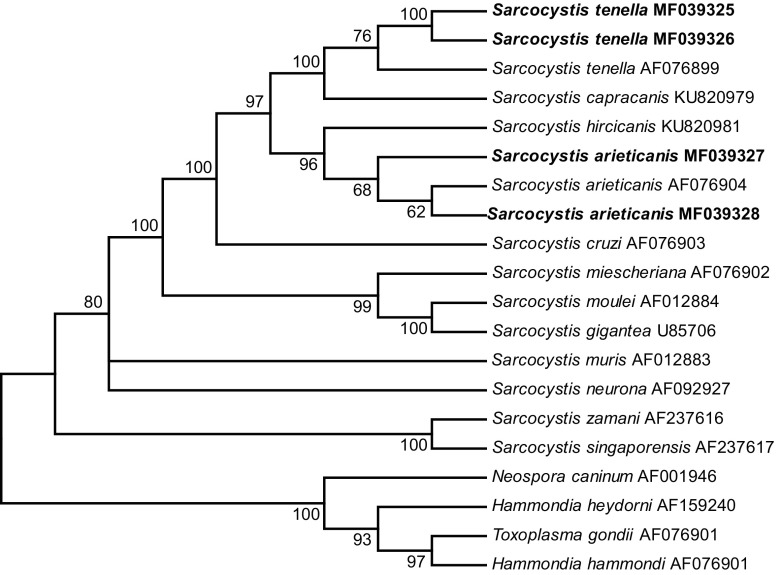
Phylogenetic tree for selected members of the Sarcocystidae based on *28S rRNA* sequences and inferred by the maximum parsimony (MP) method using the Tree-Bisection-Regrafting (TBR) algorithm. The analysis involved 20 nucleotide sequences (GenBank accession numbers behind the taxon names), and a total of 4,517 positions in the final dataset. The values between the branches represent percent bootstrap value per 1,000 replicates, and the values below 50% are not shown. The four new sequences of *Sarcocystis tenella* (MF039325 and MF039326) and *S*. *arieticanis* (MF039327 and MF039328) have the taxon name in boldface.

**Figure 5.**
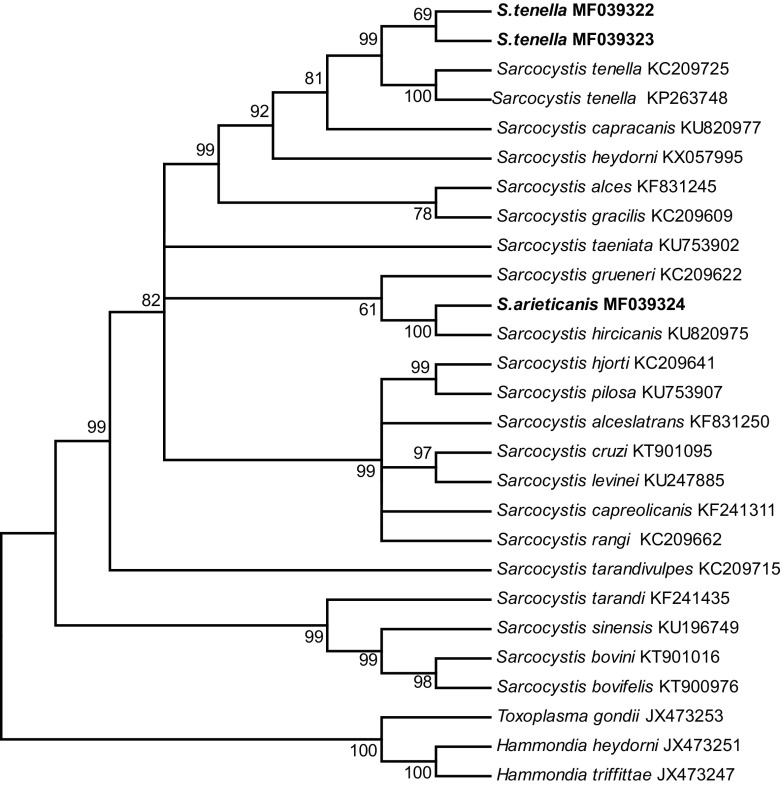
Phylogenetic tree for selected members of the Sarcocystidae based on *cox1* sequences and inferred by the maximum parsimony (MP) method using the Tree-Bisection-Regrafting (TBR) algorithm. The analysis involved 27 nucleotide sequences (GenBank accession numbers behind the taxon names), and a total of 998 positions in the final dataset. The values between the branches represent percent bootstrap value per 1,000 replicates, and the values below 50% are not shown. The three new sequences of *Sarcocystis tenella* (MF039322 and MF039323) and *S*. *arieticanis* (MF039324) have the taxon name in boldface.

The phylogenetic analyses based on either the *18S rRNA*, *28S rRNA*, or *cox1* sequences all clustered the new sequences of *S*. *tenella* and *S*. *arieticanis* within a clade comprising species with canines as the known, or presumed, definitive hosts (Figs. 3–5).

## Discussion

*Sarcocystis* spp. are among the most common parasites in domestic ruminants, and some of them can generate significant economic losses when causing clinical and subclinical disease. Up to now, at least six species of *Sarcocystis* have been named in sheep; however, only four species, i.e., *S*. *tenella*, *S*. *arieticanis*, *S*. *gigantea*, and *S*. *medusiformis*, have been frequently found in different areas, especially in Asia and Latin America [2]. In the present study, sarcocysts were common in sheep (91.9%), but only microscopic sarcocysts for *S*. *tenella* and *S*. *arieticanis* were found, and both parasites were transmitted by canids, which reflects the significant role played by dogs, rather than cats, in the transmission of these parasites in the area.

In the present study, the sarcocyst wall ultrastructures of *S. tenella* and *S*. *arieticanis* belong to “type 14” and “type 7”, respectively, using the classification by Dubey et al. (2016) [2]. *Sarcocystis capracanis* in goats is a sibling species of *S. tenella*, and the two species appear similar by LM. However, under TEM, there are some differences in sarcocyst structure, i.e., the presence (*S*. *tenella*) or absence (*S*. *capracanis*) of disk-like condensations in the apical of the villar protrusions, and the presence (*S*. *capracanis*) or absence (*S*. *tenella*) of vesicles at the base of the villar protrusions [9]. *Sarcocystis tenella*-like sarcocysts have also been described from wild Caprinae, i.e., *Sarcocystis* sp. in wild sheep (*Ovis musimon*) [13] and *Sarcocystis* sp. in chamois [14]. The ultrastructure of *S*. *arieticanis* cysts is similar to those of *Sarcocystis* spp. in different Caprinae. For example, *S*. *arieticanis* in wild sheep (*O*. *musimon*) [16], *S*. *hircicanis* in goats [9], *S*. *hircicanis*/*arieticanis*-like in blue sheep (*Pseudios nayaur*), Japanese serow (*Capricornis crispus*), and muskox (*Ovibos moschatus*) [15], plus *S*. *arieticanis*-like in Alpine ibex (*Capra ibex*) [1].

Thus, the two sarcocyst TEM types (14 and 7) in domestic sheep are common in the tissues of different, but closely related, ruminant animals; however, the relationships between these morphologically similar *Sarcocystis* spp. are not very clear. It is not easy to complete cross-transmission for *Sarcocystis* spp. using large experimental animals in the laboratory. Accordingly, characterizing sequences of different genetic markers should be a useful tool to differentiate these species of *Sarcocystis* or delineate their phylogenetic relationships. For example, *Sarcocystis* sp. in chamois has been designated as *S*. *tenella* based on the similarities of their *18S rRNA* and *cox1* sequences [10].

In the present study, four genetic markers (*18S rRNA*, *28S rRNA*, *cox1*, and *ITS-1*) for *Sarcocystis* spp. in sheep were sequenced and characterized. Among them, sequences of *cox1* for *S. arieticanis* and *ITS-1* for *Sarcocystis* spp. in sheep were the first records in GenBank. When blasting these sequences in GenBank, sequences of *18S rRNA*, *28S rRNA*, and *cox1* for *S*. *tenella* shared high identities with those of *S. capracanis*, i.e., 99.0%, 98.3%, and 93.6%, respectively; *S. hircicanis* shared high identities with *S. arieticanis*, i.e., 98.5%, 96.5%, and 92.5%, respectively. Therefore, mitochondrial *cox1* seemed to perform better than *18S rRNA* and *28S rRNA* for distinguishing *S*. *tenella* from *S*. *capracanis*, and *S. arieticanis* from *S. hircicanis*. The high identity (99.0%) between *18S rRNA* sequences for *S*. *tenella* and *S. capracanis* has even led to a debate whether sheep and goat can harbor the same *Sarcocystis* species [3, 5]. Blast search suggested that no sequences in GenBank had significant similarity with the *ITS-1* regions of *S*. *tenella* and *S. arieticanis*. However, when comparing the new sequences of the four genetic markers (*18S rRNA*, *28S rRNA*, *cox1*, and *ITS-1*) for *S*. *tenella* and *S. arieticanis*, the sequence identities were 96.3%, 95.4%, 82.5%, and 66.2%, respectively. Thus, the *ITS-1* region could be more useful for discriminating closely related *Sarcocystis* spp. because of its high divergence.
